# Subtype-specific risk models for accurately predicting the prognosis of breast cancer using differentially expressed autophagy-related genes

**DOI:** 10.18632/aging.103437

**Published:** 2020-07-10

**Authors:** Baoai Han, He Zhang, Yuying Zhu, Xingxing Han, Zhiyong Wang, Zicong Gao, Yue Yuan, Ruinan Tian, Fei Zhang, Ruifang Niu

**Affiliations:** 1 Public Laboratory, Tianjin Medical University Cancer Institute and Hospital, National Clinical Research Center for Cancer, Tianjin 300060, China; 2 Key Laboratory of Cancer Prevention and Therapy, Tianjin 300060, China; 3 Tianjin's Clinical Research Center for Cancer, Tianjin 300060, China; 4 Key Laboratory of Breast Cancer Prevention and Therapy, Ministry of Education, Tianjin 300060, China

**Keywords:** breast cancer, autophagy, TCGA database, prognosis, autophagy related genes

## Abstract

Emerging evidence suggests that the dysregulation of autophagy-related genes (ARGs) is coupled with the carcinogenesis and progression of breast cancer (BRCA). We constructed three subtype-specific risk models using differentially expressed ARGs. In Luminal, Her-2, and Basal-like BRCA, four- (*BIRC5*, *PARP1*, *ATG9B*, and *TP63*), three- (*ITPR1*, *CCL2*, and *GAPDH*), and five-gene (*PRKN*, *FOS*, *BAX*, *IFNG*, and *EIF4EBP1*) risk models were identified, which all have a receiver operating characteristic > 0.65 in the training and testing dataset. Multivariable Cox analysis showed that those risk models can accurately and independently predict the overall survival of BRCA patients. Comprehensive analysis showed that the 12 identified ARGs were correlated with the overall survival of BRCA patients; six of the ARGs (*PARP1*, *TP63*, *CCL2*, *GAPDH*, *FOS*, and *EIF4EBP1*) were differentially expressed between BRCA and normal breast tissue at the protein level. In addition, the 12 identified ARGs were highly interconnected and displayed high frequency of copy number variation in BRCA samples. Gene set enrichment analysis suggested that the deactivation of the immune system was the important driving force for the progression of Basal-like BRCA. This study demonstrated that the 12 ARG signatures were potential multi-dimensional biomarkers for the diagnosis, prognosis, and treatment of BRCA.

## INTRODUCTION

Breast cancer is a common malignant tumor in female, which occurs frequently in middle-aged and old women [1]. Nowadays, the incidence of breast cancer is increasing particularly in young adult population [2]. With the continuous development of modern medicine, the treatment of breast cancer has become more effective. However, the recurrence and metastasis of breast cancer are still challenging, and the treatment outcome after the occurrence of metastasis is poor. The high mortality among patients with recurrent breast cancer has been recognized as the major challenge of clinical treatment [3]. Breast cancer can be divided into Luminal (Luminal A, Luminal B), Her-2, and Basal-like subtypes on the basis of the gene expression profile of 50 genes, known as PAM50 molecular intrinsic subtypes [4]. The molecular subtypes of breast cancer are related to the prognosis of patients, and such subtypes are independent risk factors for prognosis [5]. Breast cancer with different molecular subtypes has different genetic background and displays differences in both biological properties and clinical prognosis [6]. Luminal breast cancer has slow progress and poor invasion properties and shows good prognosis, whereas Basal-like and Her-2 breast cancer have strong invasion and proliferation ability and often has a poor prognosis [7]. The molecular subtypes of breast cancer also serve as indicators to guide the selection of therapeutic strategies. Therefore, the identification of new subtype-specific biomarkers is necessary for the early detection and intervention of breast cancer.

Autophagy, also known as type II programmed cell death, is an important biological process that maintains homeostasis within cells by degrading aged or damaged proteins and organelles in the lysosome [8]. Autophagy plays a dual role in the pathogenesis of many diseases, including inflammatory and neurodegenerative disorders and neoplasm [9–12]. In acute kidney injury and chronic kidney disease, autophagy can reduce the stimulation of cells to a certain extent, but it can aggravate tissue damage [13]. In the early stages of cancer, autophagy inhibits the transformation and growth of cancer cells, whereas autophagy can exaggerate the proliferation of malignant cells by degrading and recovering the components of damaged or aged organelles to meet their metabolic needs for rapid growth [14, 15]. Autophagy plays a crucial role in tumor progression.

Studies have shown that autophagy is involved in regulating the growth and development of breast cancer [16, 17]. The autophagy protein Beclin-1 serves as a tumor suppressor or tumor promoter in a context-dependent manner [18]. Autophagy-associated genes *ATG8* and *UVRAG*, which have been frequently found to be deleted or mutated in BRCA tissue, are inhibitors of tumor progression [19, 20]. Although the autophagy-related genes have played a key role in BRCA initiation and progression, the clinical relevance of these autophagy genes in different molecular subtypes of BCRA has not been discussed in detail.

In this study, we explored the prognostic significance of autophagy-related genes (ARGs) in various types of BRCA tumors (Luminal, Her-2, and Basal-like) by using high-throughput expression profiles from the TCGA databases. We constructed three subtype-specific ARG risk predicting models by first identifying the differentially expressed autophagy-related genes (DEARGs) in each type of BRCA. Then, Lasso regression and Cox regression analysis were used to optimize the models, and DEARGs related to overall survival (OS) were screened out. We used these DEARGs to establish a Cox regression model (OS model) and evaluate the specificity and sensitivity of these models using ROC curve analysis. Our data show that these subtype-specific models can accurately and independently predict the prognosis of patients. These findings also provide an effective biomarker-based multi-dimensional strategy for the prognosis of BRCA patients with different molecular subtypes.

## RESULTS

### Flowchart of this study

**Figure 1:**
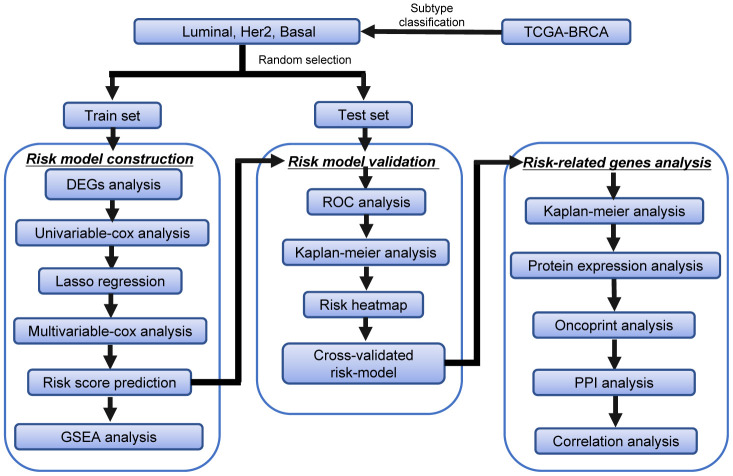
**The workflow of the identification of subtype-specific survival-related autophagy signature in BRCA.**

The detailed workflow for the construction of the subtype-specific risk models and downstream analysis was shown in Figure 1. First, we identified the DEARGs in Luminal, Her-2, and Basal-like subtypes of BRCA. Then, subtype-specific risk models were constructed using the data in the training dataset. The risk models were further verified and optimized in the testing datasets. The prediction power of these risk models was investigated by time-dependent ROC analysis. GSEA analysis was performed to analyze the differentially enriched hallmarks and KEGG pathways in the predicted high- and low-risk groups. The 12 genes in the subtype-specific risk model were subjected to Kaplan–Meier, protein expression, oncoPrint, protein–protein interaction (PPI), and correlation analyses.

### Differential expression of ARGs in Luminal, Her2, and Basal-like BRCA

**Figure 2:**
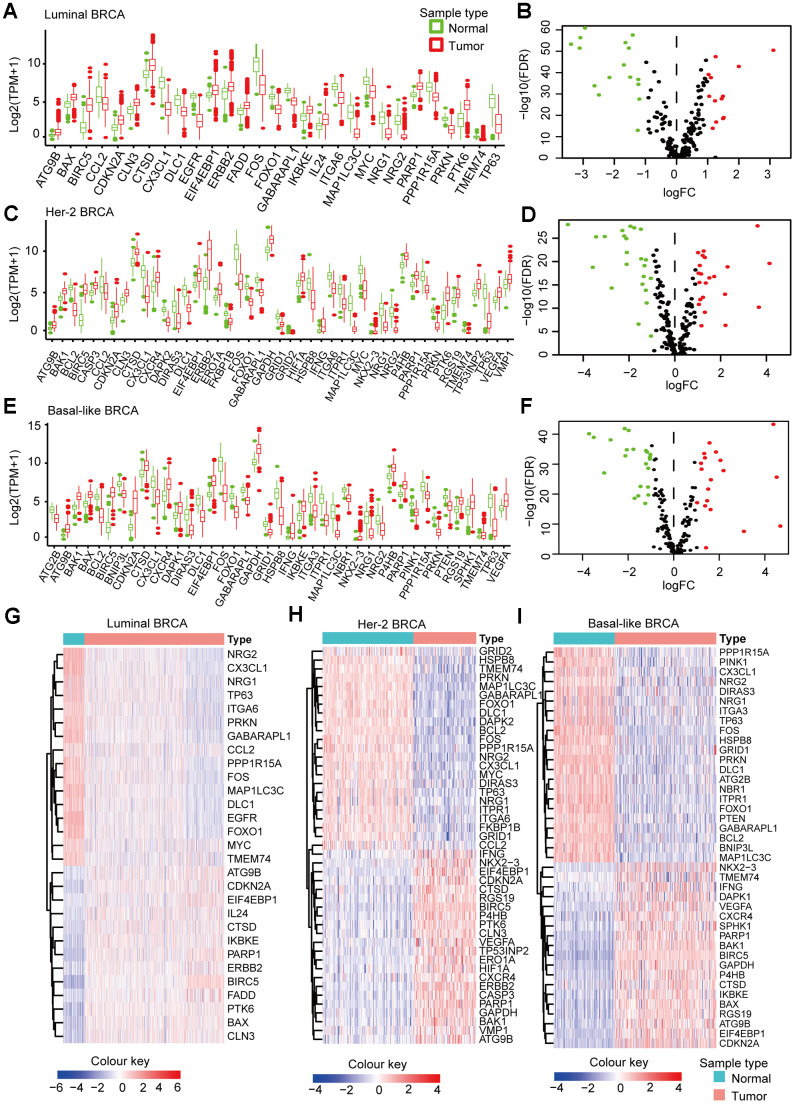
**Subtype-specific DEARGs in BRCA and normal breast tissues.** (**A**) Boxplot showing the expression pattern of DEARGs in Luminal BRCA. (**B**) Volcano plot for the 234 ARGs in Luminal BRCA. (**C**) Boxplot showing the expression pattern of DEARGs in Her-2 BRCA. (**D**) Volcano plot for the 234 ARGs in Her-2 BRCA. (**E**) Boxplot showing the expression pattern of DEARGs in Basal-like BRCA. (**F**) Volcano plot for the 234 ARGs in Basal-like BRCA. The upregulated, downregulated and no-differential expressed genes were indicated by green, red and black dots, respectively. The *P*-value was calculated by *Wilcox-test*. (**G**–**I**) Clustered heatmap of differentially expressed ARGs expression level in Luminal (**G**), Her-2 (**H**) and Basal-like (**I**) BRCA.

We downloaded the mRNA expression data and clinical information of 1109 BRCA tissue samples and 113 non-tumor samples from the TCGA database (Table 1). After extracting the expression values of 234 ARGs from the three breast cancer subtypes, we obtained the DEARGs and showed the expression patterns of the DEARGs in BRCA and non-tumor tissues by volcano plots and box plots (Figure 2). In Luminal BRCA, 29 differentially expressed genes were identified, among which, 16 and 13 genes were downregulated and upregulated (Figure 2A, 2B, 2G) in tumor tissue, respectively. In Her-2 BRCA, 45 differentially expressed genes were discovered, 23 and 22 of which were downregulated and upregulated in tumor tissue, respectively (Figure 2C, 2D, 2H). We also identified 41 differentially expressed genes in Basal-like BRCA, among them, 22 and 19 genes were upregulated and downregulated in tumor tissue (Figure 2E, 2F, 2I), respectively. In addition, we found that 18 DEARGs were shared by Luminal, Her-2, and Basal-like BRCA (Supplementary Figure 1A).

**Table 1:** Clinicopathological parameters of BRCA patients in the TCGA database.

**Clinical parameters**	**Variables**	**Total(988)**	**Percentages(%)**
Age	<=65	698	70.65%
	>65	290	29.35%
Pathological stage	Stage I	167	16.90%
	Stage II	570	57.69%
	Stage III	220	22.27%
	Stage IV	14	1.42%
	Stage X	17	1.72%
T	T1	256	25.91%
	T2	579	58.60%
	T3	116	11.74%
	T4	35	3.54%
	TX	2	0.20%
N	N0	472	47.77%
	N1	326	33.00%
	N2	110	11.13%
	N3	63	6.38%
	NX	17	1.72%
Molecular subtypes	Luminal	729	73.79%
	Her-2	78	7.89%
	Basal-like	181	18.32%
Survival status	Dead	137	13.87%
	Alive	851	86.13%

Abbreviations: T, Tumor; N, Node (lymph node regional)

### Functional annotation of the DEARGs

**Figure 3:**
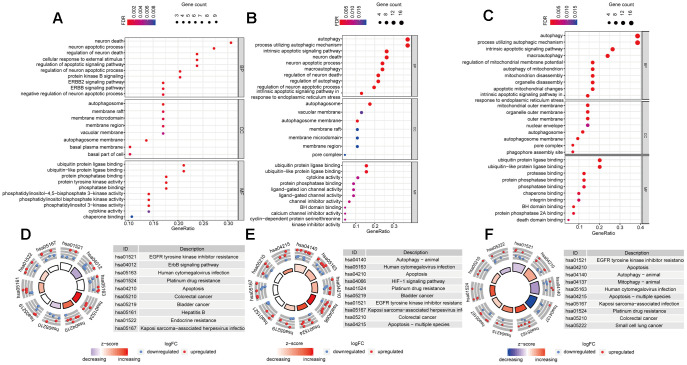
**Gene-Ontology and KEGG enrichment analysis of DEARGs.** (**A**–**C**) Gene-Ontology analysis of DEARGs in Luminal (**A**), Her-2 (**B**) and Basal-like (**C**) BRCA. (**D**–**F**) KEGG analysis of DEARGs Luminal (**A**), Her-2 (**B**) and Basal-like (**C**) BRCA. The outer circle shows a scatter plot for each term of the logFC of the assigned genes. Red circles display up-regulation pathways, and blue circles showing the down-regulation pathway.

Functional enrichment analysis of the DEARGs provided biological understanding of these genes. The GO terms and KEGG pathway enrichment of these genes were summarized in Figure 3. In Luminal BRCA, we found that the top enriched GO terms for biological processes were as follows: *neuron death*, *neuron apoptotic process*, *regulation of neuron death*. For cellular components, the GO terms were as follows: *autophagosome*, *membrane raft*, *membrane microdomain*. For molecular function, genes were mostly enriched in the following terms: *ubiquitin protein ligase binding*, *ubiquitin-like protein ligase binding*, and *protein phosphatase binding* (Figure 3A). In Her-2 BRCA, we found that the top enriched GO terms for biological processes were as follows: *autophagy*, *process utilizing autophagic mechanism*, *intrinsic apoptotic signaling pathway*. For cellular components, the GO terms were as follows: *autophagosome*, *autophagosome membrane*, *vacuolar membrane*. For molecular function, genes were mostly enriched in the following terms: *ubiquitin protein ligase binding*, *ubiquitin-like protein ligase binding*, *cytokine activity* (Figure 3B). In Basal-like BRCA, we found that the top enriched GO terms for biological processes were as follows: *autophagy*, *process utilizing autophagic mechanism*, *intrinsic apoptotic signaling pathway*. For cellular components, the GO terms were as follows: *mitochondrial outer membrane*, *organelle outer membrane*, *outer membrane*. For molecular function, genes were mostly enriched in the following terms: *ubiquitin protein ligase binding*, *ubiquitin-like protein ligase binding*, *protease binding* (Figure 3C).

In addition, in the KEGG pathway enrichment analysis for the DEARGs, these genes were associated with *EGFR tyrosine kinase inhibitor resistance*, *ERBB signaling pathway*, and *Human cytomegalovirus infection* in Luminal BRCA (Figure 3D). Terms such as *Human cytomegalovirus infection* and *Apoptosis* were enriched in Her-2 BRCA (Figure 3E). In Basal-like BRCA, terms such as *EGFR tyrosine kinase inhibitor resistance* and *Apoptosis* were significantly enriched (Figure 3F). The PPI analysis using STRING showed that the 18 shared DEARGs (Supplementary Figure 1A) were highly interconnected with PPI *P*-value < 0.01 (Supplementary Figure 1B). Functional enrichment analysis also suggested that the shared DEARGs were related to *mitochondria disassembly*, *organelle disassembly*, and *apoptosis* (Supplementary Figure 1C).

### Construction and validation of subtype-specific prognostic risk models for BRCA

**Figure 4:**
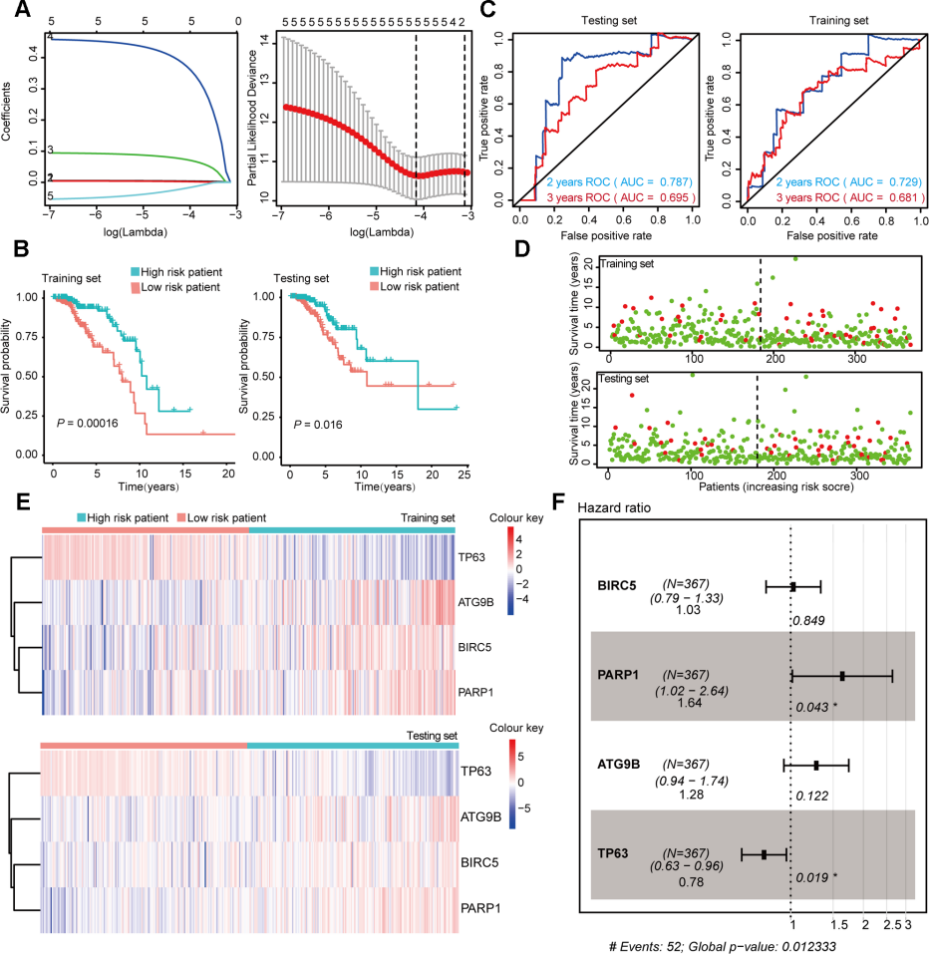
**Construction and Validation of the prognostic risk model in Luminal BRCA patients.** (**A**) Lasso regression analyses of DEARGs using the OS model. The Lasso regression was performed using prognosis-significant DEARGs in the training dataset of Luminal BRCA. (**B**) Kaplan-Meier plot represents that patients in the high-risk group had a significantly shorter overall survival time than those in the low-risk group. left, training dataset, right, testing dataset. (**C**) Time-dependent ROC curve analyses showing AUC values for OS in BRCA patients. Left, training dataset, right, testing dataset. (**D**) Dot plots showing the survival time and risk score in training set and testing set. (**E**) The heatmap of the 4 key genes expression profiles in the training dataset and testing dataset. (**F**) Forest plot showing the multivariable Cox regression analysis of 4 key genes in risk-model.

**Figure 5:**
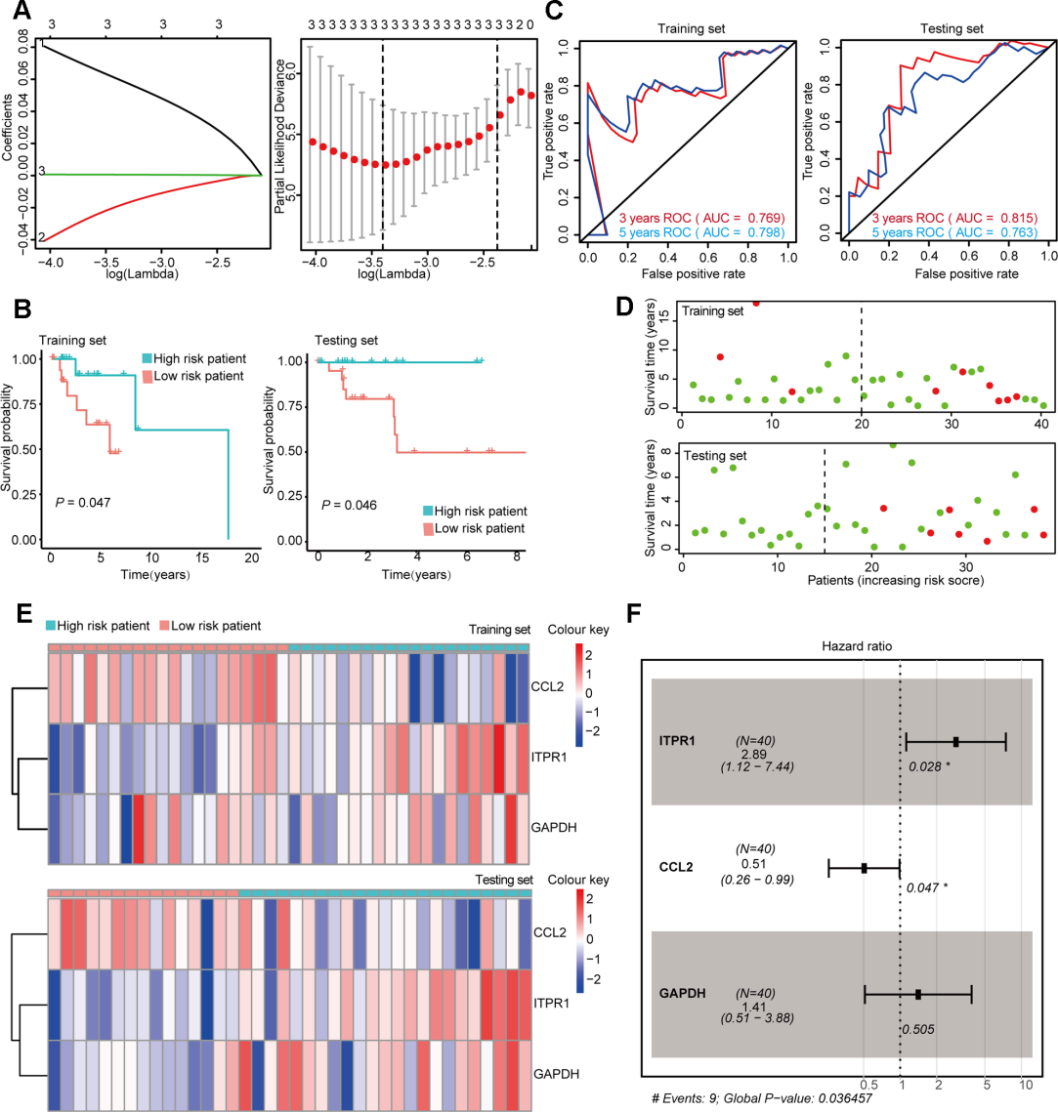
**Construction and Validation of the prognostic risk model in Her-2 BRCA patients.** (**A**) Lasso regression analyses of DEARGs using the OS model. The Lasso regression was performed using prognosis-significant DEARGs in the training dataset of Her-2 BRCA. (**B**) Kaplan-Meier plot represents that patients in the high-risk group had a significantly shorter overall survival time than those in the low-risk group. left, training dataset, right, testing dataset. (**C**) Time-dependent ROC curve analyses showing AUC values for OS in BRCA patients. Left, training dataset, right, testing dataset. (**D**) Dot plots showing the survival time and risk score in training set and testing set. (**E**) The heatmap of the 3 key genes expression profiles in the training dataset and testing dataset. (**F**) Forest plot showing the multivariable Cox regression analysis of 4 key genes in risk-model.

**Figure 6:**
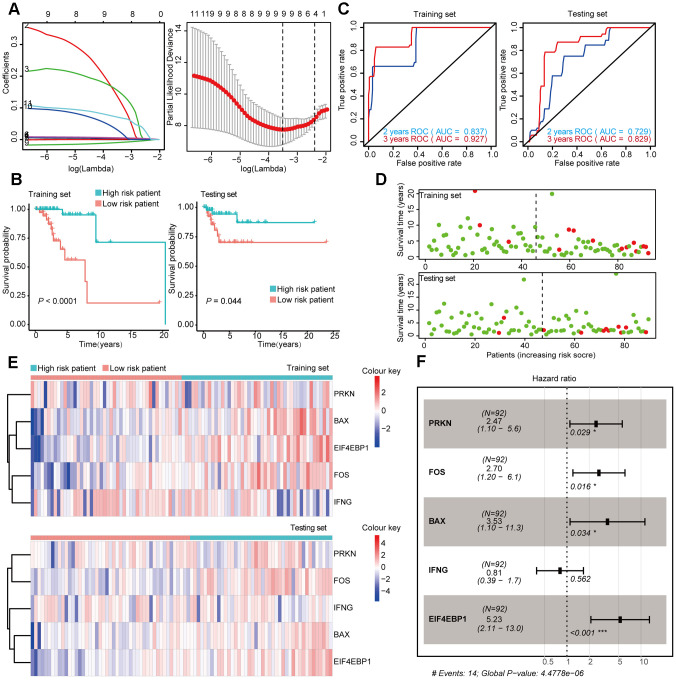
**Construction and Validation of the prognostic risk model Basal-like BRCA patients.** (**A**) Lasso regression analyses of DEARGs using the OS model. The Lasso regression was performed using prognosis-significant DEARGs in the training dataset of Basal-like BRCA. (**B**) Kaplan-Meier plot represents that patients in the high-risk group had a significantly shorter overall survival time than those in the low-risk group. left, training dataset, right, testing dataset. (**C**) Time-dependent ROC curve analyses showing the AUC values for OS in BRCA patients. Left, training dataset, right, testing dataset. (**D**) Dot plots showing the survival time and risk score in training set and testing set. (**E**) The heatmap of the 5 key genes expression profiles in the training dataset and testing dataset. (**F**) Forest plot showing the multivariable Cox regression analysis of 4 key genes in risk-model.

To explore the connection between ARGs and prognosis, we constructed risk models in Luminal, Her-2, and Basal-like breast cancer patients. Initially, univariable Cox regression analysis was performed to obtain the genes that were significantly correlated to prognosis, and then the lasso regression and multivariable Cox regression were adopted to generate the final prognostic model (Table 2, Figure 4A, 5A, 6A).

**Table 2:** The 12 selected autophagy-related genes.

**Subtypes**	**Gene**	**Coef**	**HR**	**HR.95L**	**HR.95H**	***P* value**
Luminal	BIRC5	0.03	1.03	0.79	1.33	0.85
	PARP1	0.49	1.64	1.02	2.64	0.04
	ATG9B	0.24	1.28	0.94	1.74	0.12
	TP63	-0.25	0.78	0.63	0.96	0.02
						
Her-2	ITPR1	1.06	2.89	1.12	7.44	0.03
	CCL2	-0.68	0.51	0.26	0.99	0.04
	GAPDH	0.34	1.41	0.51	3.88	0.50
						
Basal-like	PRKN	0.91	2.47	1.10	5.58	0.03
	FOS	0.99	2.70	1.20	6.07	0.02
	BAX	1.26	3.53	1.10	11.30	0.03
	IFNG	-0.22	0.81	0.39	1.67	0.56
	EIF4EBP1	1.65	5.23	2.11	12.99	<0.001

Abbreviations: HR, hazard ratio; HR.95 L/H, 95 % confidence interval of the hazard ratio.

After the construction of the subtype-specific risk models, patients were grouped into high- and low-risk groups, and then Kaplan–Meier survival analysis was performed in training and testing sets. The results showed that patients with a high risk score have a significantly poor overall survival time compared with patients with a low risk score in Luminal, Her-2, and Basal-like datasets (Figure 4B, 5B, and 6B). In addition, time-dependent ROC analysis indicated that both models can accurately predict the OS of BRCA patients (Figure 4C, 5C, and 6C).

In Luminal BRCA patients, a four-gene model (*BIRC5*, *PARP1*, *ATG9B*, and *TP63*) was successfully obtained with good prediction power for 2 year survival (area under the curve [AUC] = 0.787 in the training set and AUC = 0.729 in the testing set). The prediction accuracy for 3 year survival of this model was relatively low but acceptable (AUC = 0.695 in the training set and AUC = 0.681 in the testing set). The risk score of each patient was predicted using this model, and we identified that *BIRC5*, *PARP1*, and *ATG9B* were positive risk-correlated genes, whereas *TP63* was negative risk-correlated genes (Figure 4E, 4F).

The same data processing and analysis were also performed in other BRCA subtypes. We identified a three-gene risk model (*ITPR1*, *CCL2*, and *GAPDH*) in Her-2 BRCA with good predicting power for both 2 and 3 year survival (AUC > 0.700). The risk scores were then calculated, and *ITPR1* and *GAPDH* were identified as positive risk-correlated genes, and *CCL2* was identified as a negative risk-correlated gene (Figure 5E, 5F).

Moreover, we constructed a five-gene risk model (*PRKN*, *FOS*, *BAX*, *IFNG*, and *EIF4EBP1*) in patients with Basal-like BRCA, which has a good predicting power for 2 year survival (AUC = 0.837 in the training set and AUC = 0.729 in the testing set). The predicting power of this model for 3 year survival was good but might have mild overfitting problem (AUC = 0.927 in the training set and AUC = 0.829 in the testing set). *PRKN*, *FOS*, *BAX*, and *EIF4EBP1* were identified as positive risk-correlated genes, whereas *IFNG* was identified as a negative risk-correlated gene (Figure 6E, 6F).

### Prognostic risk models were independently related to OS in BRCA patients

We used univariable Cox regression and multivariable Cox regression to analyze the correlation among clinical parameters, such as age, histological grade, pathological stage, risk score, and OS in BRCA patients.

**Figure 7:**
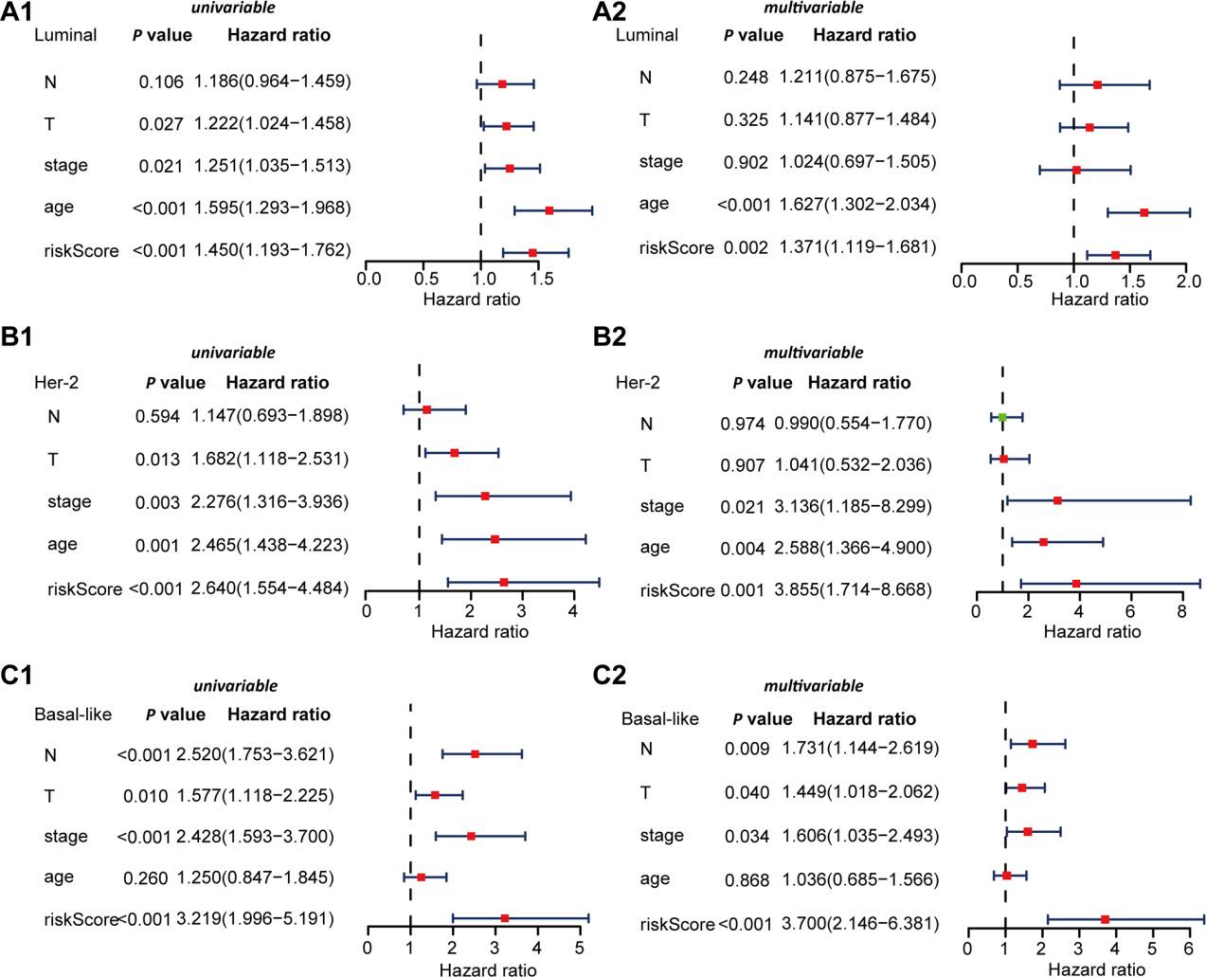
**Univariable and multivariable Cox regression analyses of OS in Luminal, Her-2, and Basal-like BRCA.** (**A**) Forest plots showing the univariable (A1) and multivariable cox regression analyses (A2) of OS in Luminal BRCA. (**B**) Forest plots showing the univariate (B1) and multivariable cox regression analyses (B2) of OS in Her-2 BRCA. (**C**) Forest plots showing the univariable (C1) and multivariable cox regression analyses (C2) of OS in Basal-like BRCA.

In Luminal BRCA, univariable Cox regression analysis showed that the age, stage, pathological stage T, and risk score were correlated with OS in BRCA patients (*P* < 0.05). Multivariable Cox analysis showed that age and risk score were correlated with OS in BRCA patients (*P* < 0.05) (Figure 7A). In Her-2 BRCA, univariable Cox regression analysis showed that age, stage, pathological stage T, and risk score were related to OS in BRCA patients (*P* < 0.05). Multivariable Cox analysis showed that age, stage, and risk score were correlated with OS in BRCA patients (*P* < 0.05) (Figure 7B). In Basal BRCA, univariable Cox regression analysis showed that stage, pathological stage T, N, and risk score were correlated with OS of BRCA patients (*P* < 0.05). Multivariable Cox analysis showed that stage, pathological stage T, N, and risk score were significantly correlated with OS (*P* < 0.05) (Figure 7C). These results indicated that the identified subtype-specific prognostic models can be used independently to predict OS in BRCA patients with different molecular subtypes.

### Comprehensive analysis of genes in subtype-specific autophagy prognostic models

**Figure 8:**
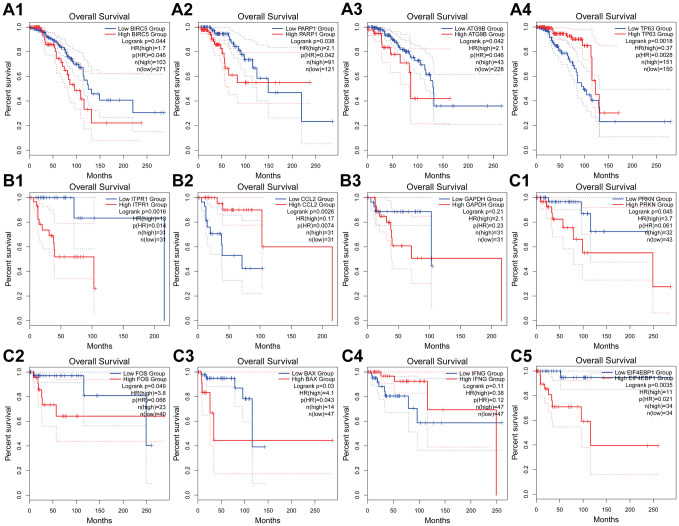
**Kaplan-Meier analyses of ARGs in subtype-specific prognosis models.** (**A**) Kaplan-Meier analyses of *BIRC5*, PARP1, *ATG9B* and *TP63* in Luminal BRCA. (**B**) Kaplan-Meier analyses of *ITPR1*, *CCL2* and *GAPHD* in Her-2 BRCA. (**C**) Kaplan-Meier analyses of *PRKN*, *FOS*, *BAX*, *INFG* and *EIF4EBP1* in Basal-like BRCA. The statistical significance was determined by *Log-rank* test. The dashed lines represent 95% confidence interval.

We obtained a total of 12 genes in the subtype-specific risk models, and then we further evaluated the prognostic value of the selected genes in other databases. The genes were subjected to *GEPIA* database to perform the Kaplan–Meier analysis. The results showed that in Luminal BRCA, *BIRC5*, *PARP1*, and *ATG9B* negatively correlated with OS, whereas the high expression of *TP63* indicated a good prognosis (Figure 8A1–4). In Her-2 BRCA, *ITPR1* and *GAPDH* were correlated with bad prognosis, whereas *CCL2* was a good prognostic marker (Figure 8B 1–3). In Basal BRCA, the high expression of *PRKN*, *FOX*, *BAX*, and *EIF4EBP1* indicated a bad prognosis; however, *INFG* was a protective molecule that significantly correlated with good prognosis (Figure 8C 1–5). Collectively, the results of Kaplan–Meier analysis were consistent with the results of univariable Cox analysis, which indicated that all genes have been inculcated in the risk-specific models and have a good prognostic predicting power.

**Figure 9:**
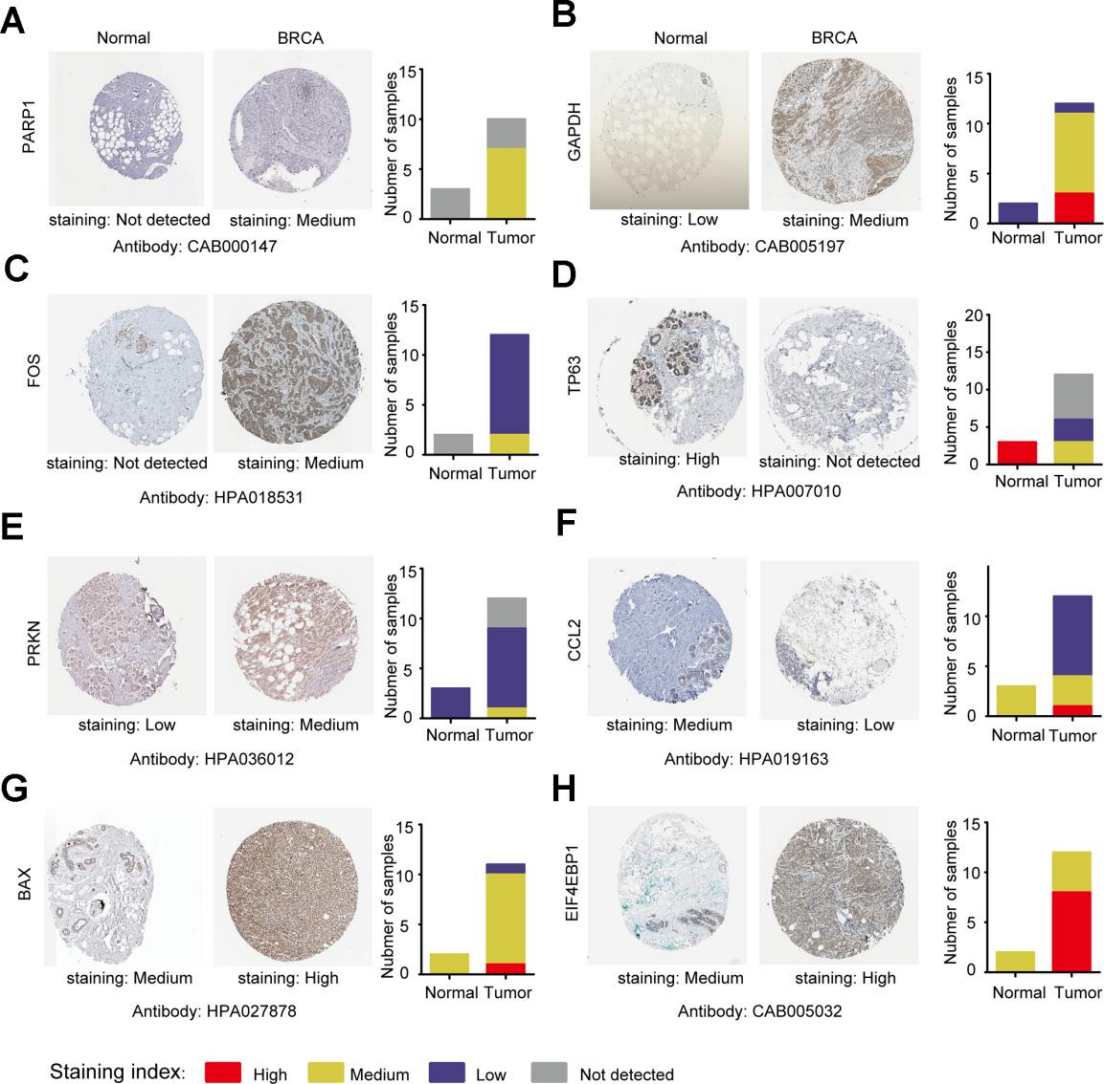
**Analysis of the protein expression of ARGs in subtype-specific prognostic models by HPA.** (**A**–**H**) The protein expression of *PARP1*, *GAPDH*, *FOS*, *TP63*, *PRKN*, *CCL2*, *BAX* and *EIF4EBP* were determined by immunohistochemistry using indicated antibodies in HPA database, the staining strengths were annotated as Not detected, Low, Medium and High. The bar plots indicating the number of samples with different staining strength in HPA database.

Next, we analyzed the protein expression patterns of the genes in subtype-specific risk models by the HPA database (Figure 9). The results showed that PARP1, GAPDH, and FOS protein were less expressed in normal breast tissues but were moderately expressed in breast cancer tissues (Figure 9A–9C). TP63 was highly expressed in normal tissues but not detected in tumor tissues (Figure 9D). The CCL2 protein was moderately expressed in normal tissues and lowly expressed in cancer tissues (Figure 9F). The EIF4EBP1 protein was moderately expressed in normal tissues and highly expressed in cancer tissues (Figure 9H). These results were consistent with most of our previous mRNA level observations. However, we found that PRKN and BAX protein were only slightly upregulated in cancer tissues (Figure 9E, 9G).

**Figure 10:**
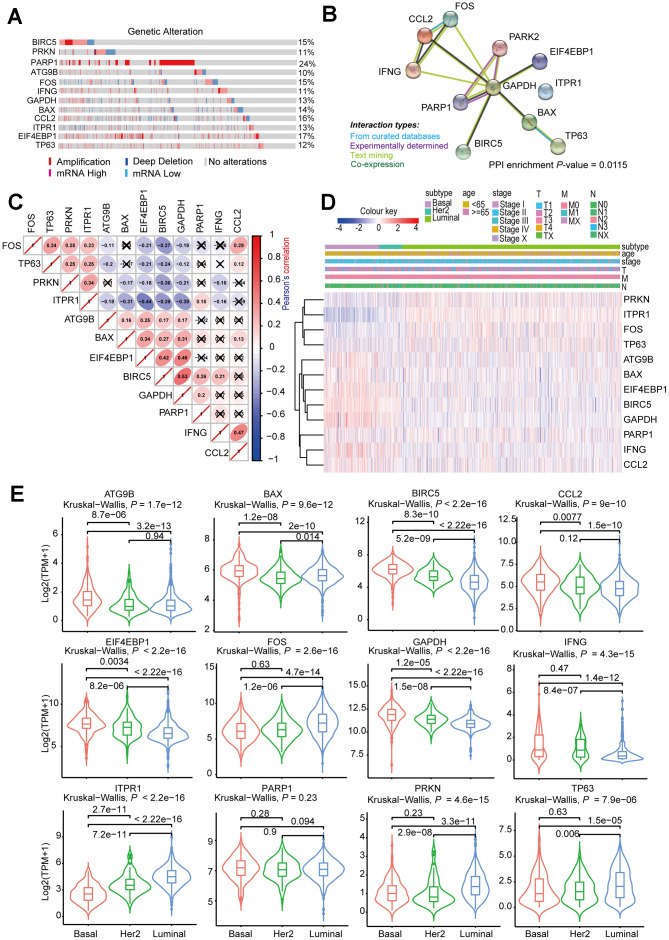
**Comprehensive analysis of ARGs in subtype-specific prognostic models.** (**A**) OncoPrint showing the copy number alterations and mRNA expression alterations of 12 ARGs in subtype-specific autophagy prognostic models. The analysis was performed by *cBioProtal* database using *MATABRIC* dataset. (**B**) Protein-protein interaction analysis of genes in subtype-specific autophagy prognostic model by *STRING* database. (**C**) Clustered heatmap showing the correlation of genes expression in subtype-specific autophagy prognostic model. The correlation was calculated by Pearson’s correlation using log2 (TPM+1). Not statistically significant correlations were defined as *P* > 0.05 and marked by a black cross. (**D**) Clustered heatmap showing the genes expression and clinical information in BRCA patients. (**E**) Violin plots showing identified gene expression in Luminal, Her-2 and Basal-like BRCA. The *P* values were calculated by *Wilcox-test* (two groups comparison) and *Kruskal-* *Wallis test* (three groups comparison).

We then investigated the CNV and mRNA expression alternation of the aforementioned genes by using the *cbioProtal* database (Figure 10A). The results indicated that CNVs contributed to the mRNA expression alterations of these analyzed genes. Notably, *PARP1*, *EIF4EBP1*, and *CCL2* showed the highest CNVs and mRNA expression alterations throughout the analyzed samples, which might indicate that the CNVs were the primary driving power response to the mRNA expression alterations of such genes. We also analyzed the PPI of the 12 selected genes, and the results suggested that such genes were highly interconnected (PPI enrichment *P* < 0.05), and *GAPDH* was the major hub gene in the PPI network (Figure 10B). The correlation analysis of the 12 selected genes was consistent with PPI analysis, which showed that most of the genes were correlated with mRNA expression (Figure 10C). The clustered heatmap showed that the 12 selected genes can be clustered into two groups, one of which showed a universal upregulation pattern in Basal-like BRCA (*IFNG*, *CCL2*, *ATG9B*, *BAX*, *EIF4EBP1*, *GAPDH*, and *BIRC5*), whereas genes in another cluster (*PRKN*, *FOS*, *TP63*, and *ITPR1*) displayed a low expression pattern in Basal-like BRCA. This finding might indicate that the two clusters of genes played different functional roles in Basal-like BRCA (Figure 10D). Consistently, the violin plots also showed that most of the selected genes were differentially expressed between Luminal, Basal-like, and Her-2 BRCA (Figure 10E).

### Mechanistic exploration of model-predicted high-risk patients by gene set enrichment analysis (GSEA)

**Figure 11:**
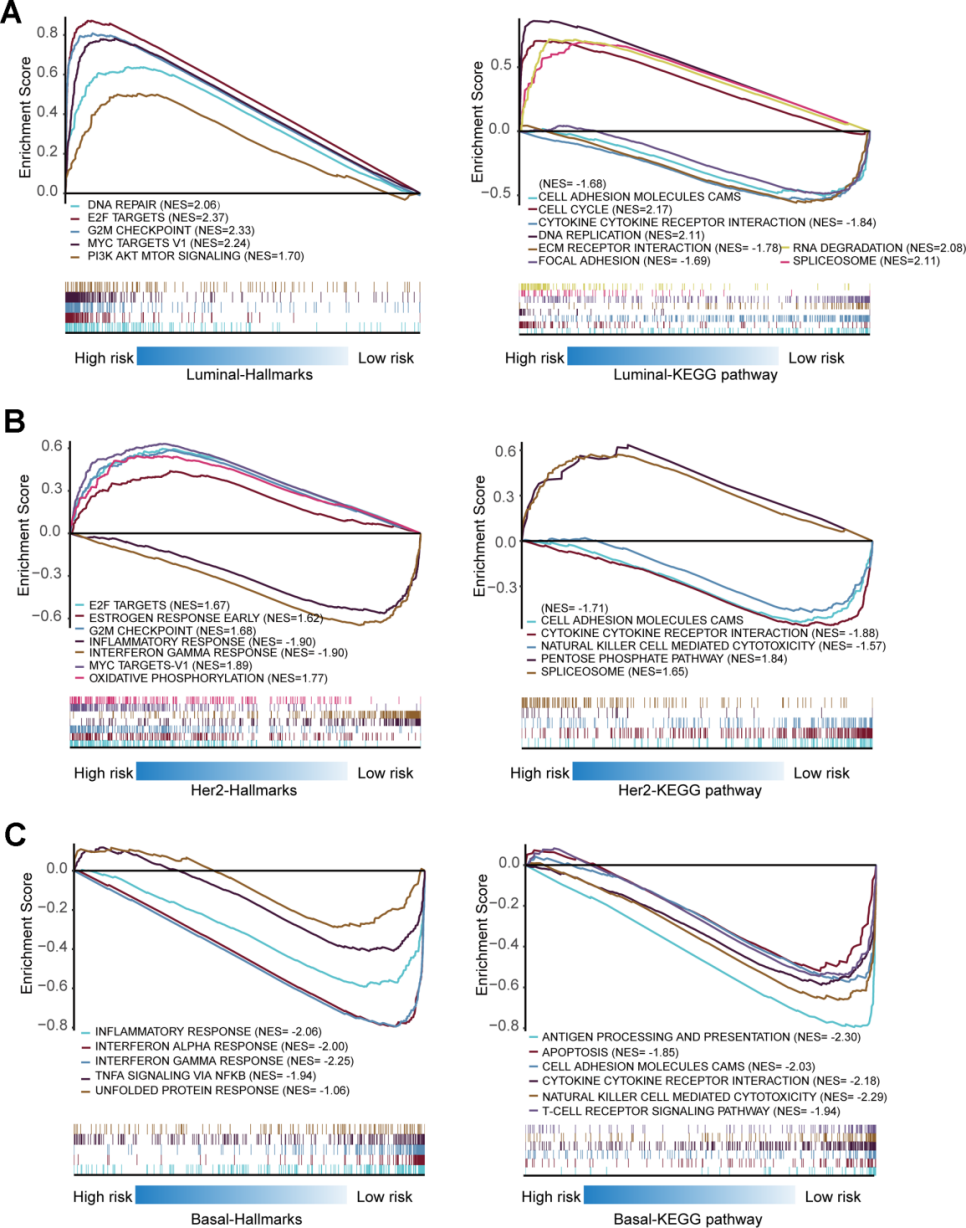
**Gene set enrichment analysis of genes in high-risk and low-risk patients in Luminal, Her-2 and Basal-like BRCA.** (**A**) Gene set enrichment analysis (GSEA) showing the enrichment of Hallmarks and KEGG pathways in high-risk and low-risk patients with Luminal BRCA. (**B**) GSEA showing the enrichment of Hallmarks and KEGG pathways in high-risk and low-risk patients with Her-2 BRCA. (**C**) GSEA showing the enrichment of Hallmarks and KEGG pathways in high-risk and low-risk patients with Basal-like BRCA.

Considering that the high- and low-risk patients had a significant prognostic difference in the OS, we explored the mechanisms that contributed to this observation by a computational approach. The GSEAs were performed in Luminal, Basal-like, and Her-2 BRCA to interpret the enriched hallmarks and pathways between high- and low-risk patients. In Luminal BRCA, the results showed that several canonical tumor-promoting molecules were upregulated in the high-risk groups, including DNA repair, E2F targets, and G2M checkpoint. Otherwise, certain well-recognized Onco-signaling pathways were highly activated in high-risk tumors, such as PI3K AKT mTOR signaling, DNA replication, and cell cycle. High enrichment of focal adhesion, cell adhesion molecules (CAMs), and ECM receptor interaction-related genes were found in low-risk tumors, which might suggest that high-level cell–cell adhesion was a protective event in Luminal BRCA (Figure 11A).

Similar to Luminal BRCA, canonical tumor-promoting molecules and pathways in Her-2 BRCA were also enriched in high-risk tumors. The low-risk tumors were enriched in terms such as cytokine–cytokine receptor interaction and natural killer cell-mediated cytotoxicity, which suggested that the activation of the immune system contributed to the restraints of tumor progression (Figure 11 B).

None of the analyzed hallmarks or KEGG pathways were enriched in Basal-like BRCAs with high risk score; however, several immune-related molecules and signaling pathways were highly enriched in low-risk patients of Basal-like BRCA, such as interferon alpha/gamma response, antigen processing and presentation, cytokine–cytokine receptor interaction, natural killer cell-mediated cytotoxicity, and T-cell receptor signaling pathway (Figure 11C). This observation indicated that the activation of the immune system was important in controlling the progression of Basal-like BRCA.

Collectively, such in silico analyses proposed that ARGs might correlate with several well-known oncogenes and/or Onco-pathways that contributed to the progression of Luminal and Her-2 BRCA, but the dysregulation of ARGs might inhibit the tumor-suppressing immune reactions that exaggerated the aggressive nature of Basal-like BRCA.

## DISCUSSION

Breast cancer is a common malignant tumor in female patients, which is one of the primary causes of death in women with malignant tumors [21, 22]. The development of a new molecularly targeted therapy is relatively slow because of limited effective molecular biomarkers for BRCA prognostic monitoring and pharmaceutical intervention [23]. At present, the role of autophagy in BRCA is controversial. Current data shows that autophagy can inhibit or promote the progression of cancer in a context-dependent manner [14]. Autophagy can also regulate the response of cancer to various therapies, which contributes to the acquisition of drug resistance in cancer cells [8]. Therefore, studying the expression pattern of ARGs is important to understand the role of autophagy in BRCA [24, 25]. Although the correlation between single ARG and BRCA has been discussed in previous studies, a comprehensive in-depth analysis of the clinical correlation between ARGs and the subtype of BRCA has not been carried out [26, 27]. In addition, the relationship between the expression of ARGs and the prognosis of BRCA patients is not clear.

**Figure 12:**
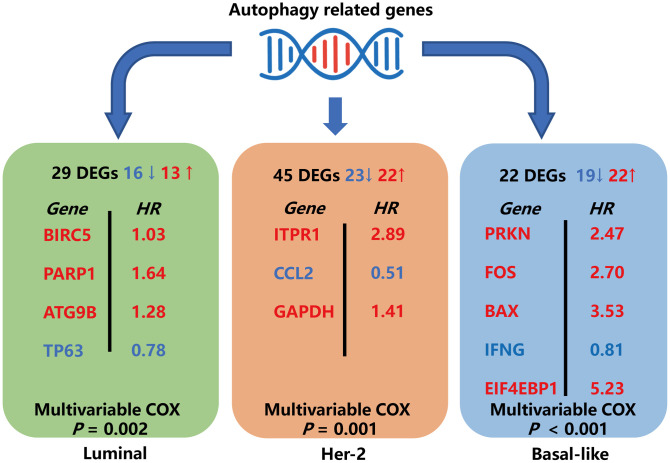
**Schematic summary diagram for the three subtype-specific risk-models constructed in this study.**

In this study, we explore the expression profile of ARGs in the TCGA database to identify molecular biomarkers related to the diagnosis, treatment, and prognosis of BRCA patients. We first screened the DEARGs between BRCA and non-tumor tissues. Considering that these genes may be deeply related to the occurrence of BRCA, we performed GO and KEGG analysis on these genes. Most of the enriched pathways are autophagy-related pathways. Some other annotations were also found, including *apoptosis signaling pathway, ERBB signaling pathways*, and *mitochondria/organelles disassembly*. For the KEGG pathway, we identified that the ERBB2 signaling pathway was enriched. The ERBB2 signal is closely related to autophagy. The activation of the ERBB2 signaling pathway can induce autophagy in a variety of cancers [28–30]. Then, we analyzed three breast cancer subtypes and constructed models by Cox regression and lasso regression, subsequently. Multivariable Cox regression analysis of the prognostic models and other clinical parameters showed that the model-calculated risk scores independently predicted the prognosis of patients with BRCA. The major findings of this study were summarized in Figure 12.

Recently, on the basis of the GEO database, Gu and his colleagues constructed an ARG model consisting of eight genes (*BCL2*, *BIRC5*, *EIF4EBP1*, *ERO1L*, *FOS*, *GAPDH*, *ITPR1*, and *VEGFA*), which can be used as an independent prognostic indicator of breast cancer [31]. Notably, five of those genes (*BIRC5*, *EIF4EBP1*, *FOS*, *GAPDH*, and *ITPR1*) are also identified in our subtype-specific risk models. Considering that the molecular background is distinct in Luminal, Her-2, and Basal-like breast cancer, this scoring system did not consider the subtype-specific genetic background, which may limit clinical application. Thus, a subtype-specific scoring system is necessary and more reliable in clinical application to predict patient’s prognosis.

In the Luminal subtype, a four-gene (*BIRC5*, *PARP1*, *ATG9B*, and *TP63*) risk model was identified. High-risk patients in this model are related to DNA repair, G2M checkpoints, MYC-related genes, and PI3K/AKT/mTOR pathways in GSEA, which indicate that tumors in the high-risk group may have higher proliferation potential. Furthermore, GESA-KEGG enrichment analysis showed that cytokine receptor and adhesion pathways were highly activated in the low-risk patients, which suggested that the activation of immune-related pathways and expression of adhesion molecules may have an inhibitory effect on tumor progression in the Luminal type. A prognostic model of three genes (*ITPR1*, *CCL2*, and *GAPDH*) was obtained in the Her-2 subtype. Similar to the Luminal type, E2F downstream genes, G2M checkpoint-related genes, and MYC target genes are highly enriched in high-risk patients. However, low-risk patients are highly enriched in CAMS and cytokine-related molecules. This finding indicates that in Her-2 BRCA, not only cell proliferation-related signals play a role in promoting the development of tumors but also the deactivation of immune-related pathways can promote the progression of tumors [32]. A prognostic model of five genes (*PRKN*, *FOS*, *BAX*, *IFNG*, and *EIF4EBP1*) was obtained in a basal-like subtype. Unlike the luminal and Her-2 subtypes, some well-known tumor-promoting molecules and signaling pathways were not enriched in high-risk patients based on the GSEA analysis. Notably, the immune-related molecules, such as immunology-related signaling, TNFα signaling, antigen presentation, natural killer cell, and T-cell receptor signaling, are highly enriched in the low-risk patients. Autophagy-related genes exert a distinct functional role in the Basal-like BRCA, which indicates that the microenvironment and immune-related signaling are related to autophagy in Basal-like BRCA [33–35].

Based on the comprehensive analysis of ARG expression profiles and corresponding clinical characteristics, three subtype-specific prognostic ARG risk models were identified. The genes identified in the aforementioned models provide new targets for the treatment and intervention of breast cancer. The primary limitation of our findings is that the data used in our study were obtained from several public databases. The 12 identified ARGs may prove new perspectives for the diagnosis, prognosis, and treatment of breast cancers. However, the clinical implication of these findings is challenging and remains unclear, and these findings need to be validated in future clinical trials. In addition, the Luminal and Basal-like risk models perform well in predicting 2 years of survival but less accurate in predicting 3 years of survival. Such limitations are probably due to limited data involved in model construction, and future studies are needed to improve the performance of such risk models by involving more data in model construction. Moreover, the mechanisms by which ARGs regulate BRCA initiation and progression require further study. Our study shows that the DEARGs have remarkable potential as biomarkers and therapeutic targets for the diagnosis and prognosis of BRCA. Further investigation is needed to confirm our findings. These models should also be verified in local clinical cohorts to improve the accuracy of the prediction.

## MATERIALS AND METHODS

### Data collection

In this study, ARGs were downloaded from human autophagy database (HADB, http://www.autophagy.lu/index.html), and clinicopathological parameters and RNA sequencing results (FPKM) of BRCA were obtained from the TCGA data portal (https://portal.gdc.cancer.gov/). The expression data were converted to TPM, and the batch effect in the data was analyzed by TCGA Batch Effects Viewer and PCA analysis. No significant batch effect was found. The PAM50 classification information of samples included in this study was downloaded from UCSC Xena (http://xena.ucsc.edu/).

### Differential expression analysis of ARGs

The Wilcox test was used to estimate the DEARGs between BRCA and non-tumor samples. Genes with at least a two fold change and corresponding *P* values of less than 0.05 were selected as ARGs with significant differential expression (DEARGs). A series of gene functional enrichment analyses was then performed to discover the primary biological characteristics of these genes. The *clusterProfiler* package in R was used to identify the enriched GO and KEGG, and the *GOplot* package of R was used to visualize the enriched items.

### Construction and validation of subtype-specific risk prediction models using DEARGs

We randomly divided the patients with complete OS information into two groups: the training group and the testing group (Table 3). We used the data from the training group to build a Cox regression model for the OS, and we used the testing group to verify the accuracy of the model. Initially, univariable Cox regression analysis was used to select potential prognostic genes. Then, the lasso regression analysis was used to eliminate false-positive parameters caused by overfitting. Finally, Cox proportional risk regression was used to establish an OS prognostic risk model.

**Table 3:** Demographics of the patients in training and testing groups.

**Subtypes**	**Clinical parameters**	**Variables**	**Training group(%)**	**Testing group(%)**
Luminal	Survival status	Dead	52(5.26)	44(4.45)
		Alive	315(31.88)	318(32.19)
				
Her-2	Survival status	Dead	9(0.91)	7(0.71)
		Alive	31(3.14)	31(3.14)
				
Basal-like	Survival status	Dead	14(1.42)	11(1.11)
		Alive	78(7.89)	78(7.89)

Total patient = 988

### Calculation of risk score

The risk score of each patient was calculated by the regression coefficient of a single gene and the expression value of each gene. The calculation formula is as follows:

$$\begin{array}{c} Riskscore\;(Patients)=\Sigma_{i=1,2,3,i}coefficient\;(ARGi)\\ \times \;expression\;(ARGi) \end{array}$$

where ARGi represents the identifier of the *i*-th selected ARG. The value of coefficient (ARGi) is the regression coefficient estimated by ARGi based on Cox proportional risk regression analysis. The risk score is a measurement of the prognostic risk of each BRCA patient. We divided the BRCA patients into high- and low-risk groups, with the median risk score of the training group as the boundary. High risk scores suggested a poor prognosis for BRCA patients.

### Comprehensive analysis of ARGs in the risk-specific model

The ARGs in risk-specific models were subjected to Kaplan–Meier analysis using the *GEPIA* database (http://gepia.cancer-pku.cn/). The log-rank test was used to determine statistical significance. The protein expression of the selected ARGs was analyzed by comparing immunohistochemistry staging images in The Human Protein Atlas database (http://www.proteinatlas.org/). The samples were annotated as not detected, low, medium, and high on the basis of the staining strength. The 12 ARGs in the risk-specific model were analyzed by using the *cbioProtal* database (http://www.cbioportal.org/) to assess the copy number variation and mRNA expression variation. The threshold to determine mRNA expression alteration was set as Z-score=1.5. For PPI network construction, the 12 selected ARGs were subjected to *STRING* database (https://string-db.org/). The interacting proteins (both experimentally determined and computationally predicted) were marked as colored lines between genes. For correlation analysis of selected ARGs, the gene expression data were extracted and logarithmically transformed. Then, Pearson’s correlation was calculated among all the gene pairs. The expression of the selected ARGs was compared among different molecular subtypes of BRCA (Luminal, Her-2, and Basal-like) using the Wilcox test and Kruskal–Wallis test, and the distribution of the gene expression was presented by violin plots.

### Gene set enrichment analysis (GSEA)

To explore the hallmarks and pathways that were enriched in the predicted high- and low-risk group, GSEA was performed. Using GSEA, the present study tested whether the activated/repressed gene signatures were enriched for high-risk vs. low-risk cases. The enrichment of pre-defined hallmarks and KEGG pathways was calculated using a normalized enrichment score (NES) and normalized *P*-value. Terms with |NES|>1 and *P*<0.05 were considered significantly enriched.

### Statistical analysis

All statistical analyses were performed using R software (version 3.6.0). *P* < 0.05 was considered statistically significant. Wilcox test or Kruskal–Wallis test was used to evaluate the distribution differences among variables. Kaplan–Meier survival curve analysis and log-rank test were used to analyze OS. The Cox regression model was used to analyze the factors influencing the survival of BRCA patients. Cox proportional risk regression model was used for univariable and multivariable analyses. Time-related ROC analysis was used to assess the accuracy of models for predicting prognosis. We used the survival time, survival state, and risk score obtained from the risk models to draw the ROC curve in the R software using the *survivalROC* package, and both 2 year and 3 year ROC curve was drawn. The AUC value greater than or equal to 0.70 was regarded as the significant prediction value, and AUC value greater than or equal to 0.65 was regarded as the acceptable prediction value.

## Supplementary Material

Supplementary Figure 1
