# Supplementary material for: Subtype-specific risk models for accurately predicting the prognosis of breast cancer using differentially expressed autophagy-related genes
Source: Aging (Albany NY). 2020 Jul 10;12(13):13318–37. doi: 10.18632/aging.103437 (PMC7377895; doi:10.18632/aging.103437)
Supplement: Supplementary Figure 1 [file aging-12-103437-s001..pdf]

SUPPLEMENTARY FIGURE

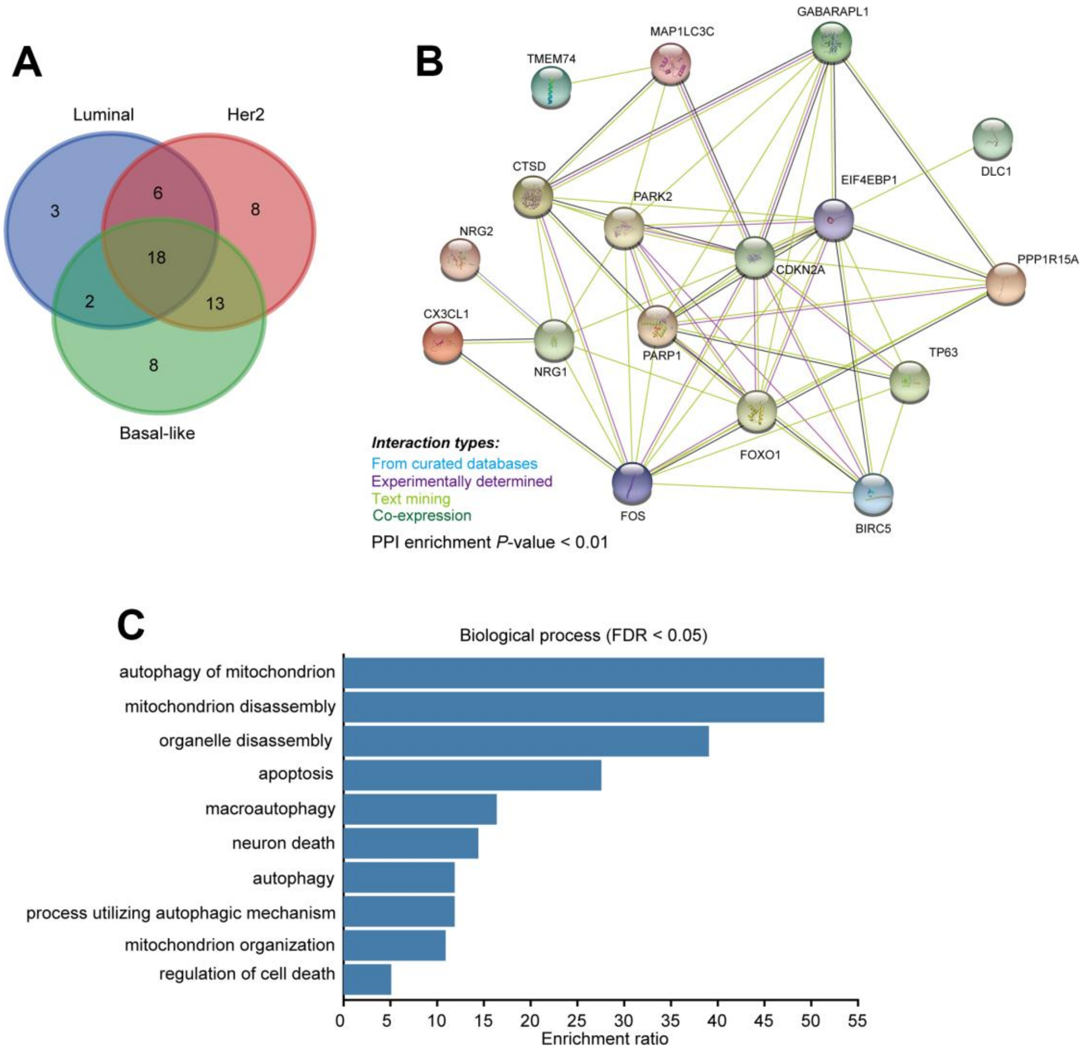

Supplementary Figure 1. Analysis of the overlapping DEARGs among the three subtypes. (**A**) Venn diagram of overlapping DEARGs between three subtypes. (**B**) PPI analysis of overlapping genes. (**C**) Gene Ontology enrichment analysis of overlapping genes.
